# Enabling a robust scalable manufacturing process for therapeutic exosomes through oncogenic immortalization of human ESC-derived MSCs

**DOI:** 10.1186/1479-5876-9-47

**Published:** 2011-04-25

**Authors:** Tian Sheng Chen, Fatih Arslan, Yijun Yin, Soon Sim Tan, Ruenn Chai Lai, Andre Boon Hwa Choo, Jayanthi Padmanabhan, Chuen Neng Lee, Dominique PV de Kleijn, Sai Kiang Lim

**Affiliations:** 1 Institute of Medical Biology, A*STAR, 8A Biomedical Grove, 138648 Singapore; 2 Laboratory of Experimental Cardiology, University Medical Centre Utrecht, Heidelberglaan 100, 3584 CX Utrecht, the Netherlands; 3 National University of Singapore, Graduate School for Integrative Sciences and Engineering, 28 Medical Drive, 117456 Singapore; 4 Bioprocessing Technology Institute, A*STAR, 20 Biopolis Way, 138671 Singapore; 5 Department of Surgery, YLL School of Medicine, NUS, 5 Lower Kent Ridge Road, 119074 Singapore; 6 Interuniversity Cardiology Institute of the Netherlands, Catharijnesingel 52, 3511 GC Utrecht, the Netherlands

## Abstract

**Background:**

Exosomes or secreted bi-lipid vesicles from human ESC-derived mesenchymal stem cells (hESC-MSCs) have been shown to reduce myocardial ischemia/reperfusion injury in animal models. However, as hESC-MSCs are not infinitely expansible, large scale production of these exosomes would require replenishment of hESC-MSC through derivation from hESCs and incur recurring costs for testing and validation of each new batch. Our aim was therefore to investigate if *MYC *immortalization of hESC-MSC would circumvent this constraint without compromising the production of therapeutically efficacious exosomes.

**Methods:**

The hESC-MSCs were transfected by lentivirus carrying a *MYC *gene. The transformed cells were analyzed for *MYC *transgene integration, transcript and protein levels, and surface markers, rate of cell cycling, telomerase activity, karyotype, genome-wide gene expression and differentiation potential. The exosomes were isolated by HPLC fractionation and tested in a mouse model of myocardial ischemia/reperfusion injury, and infarct sizes were further assessed by using Evans' blue dye injection and TTC staining.

**Results:**

*MYC*-transformed MSCs largely resembled the parental hESC-MSCs with major differences being reduced plastic adherence, faster growth, failure to senesce, increased MYC protein expression, and loss of *in vitro *adipogenic potential that technically rendered the transformed cells as non-MSCs. Unexpectedly, exosomes from *MYC*-transformed MSCs were able to reduce relative infarct size in a mouse model of myocardial ischemia/reperfusion injury indicating that the capacity for producing therapeutic exosomes was preserved.

**Conclusion:**

Our results demonstrated that *MYC *transformation is a practical strategy in ensuring an infinite supply of cells for the production of exosomes in the milligram range as either therapeutic agents or delivery vehicles. In addition, the increased proliferative rate by *MYC *transformation reduces the time for cell production and thereby reduces production costs.

## Background

Mesenchymal stem cells (MSCs) are multipotent stem cells that have a limited but robust potential to differentiate into mesenchymal cell types, e.g. adipocytes, chondrocytes and osteocytes, with negligible risk of teratoma formation. MSC transplantation has been used in clinical trials and animal models to treat musculoskeletal injuries, improve cardiac function in cardiovascular disease and ameliorate the severity of graft-versus-host-disease [1]. In recent years, MSC transplantations have demonstrated therapeutic efficacy in treating different diseases but the underlying mechanism has been controversial [2-9]. Some reports have suggested that factors secreted by MSCs were responsible for the therapeutic effect on arteriogenesis, stem cell crypt in the intestine, ischemic injury, and hematopoiesis [9-20]. In support of this paracrine hypothesis, many studies have observed that MSCs secrete cytokines, chemokines and growth factors that could potentially repair injured cardiac tissue mainly through cardiac and vascular tissue growth and regeneration [21,22]. This paracrine hypothesis could potentially provide for a non-cell based alternative for using MSC in treatment of cardiovascular disease [23]. Non-cell based therapies as opposed to cell-based therapies are generally easier to manufacture and are safer as they are non-viable and do not elicit immune rejection.

We have previously demonstrated that culture medium conditioned by MSCs that were derived from human embryonic stem cells (HuES9E1 MSCs) or fetal tissues could protect the heart from myocardial ischemia/reperfusion injury and reduce infarct size in both pig and mouse models of myocardial ischemia/reperfusion (MI/R) injury [24-27]. Subsequent studies demonstrated that this cardioprotection was mediated by exosomes or microparticles of about 50-100 ηm in diameter and these microparticles carry both protein and RNA load [24-28]. These exosomes could be purified as a population of homogenously sized particles by size exclusion on HPLC and reduced infarct size in a mouse model of MI/R injury at about a tenth of the dosage of the conditioned medium [24,25].

The identification of exosomes as the therapeutic agent in the MSC secretion could potentially provides for a biologic-rather than cell-based treatment modality. Unlike cells, exosomes do not elicit acute immune rejection and being non-viable and much smaller, they pose less safety risks such as the formation of tumor or embolism. Furthermore unlike cell-based therapies where there is a need to maintain viability, manufacture and storage of non-viable exosomes is less complex and therefore less costly. Besides being therapeutic agents, exosomes have been advocated as "natural" drug delivery vehicles [29]. These lipid vesicles could be loaded with therapeutic agents and be used to deliver the agents in a cell type specific manner. hESC-MSCs could be the ideal cellular source for the efficient production of exosomes. We have demonstrated that these cells could be grown in a chemically defined medium during the production and harvest of exosomes and these exosomes could be purified by HPLC to generate a population of homogenously sized particles [27]. Another advantage is that these cells were derived from hESC, an infinitely expansible cell source.

While hESC-MSCs are also highly expansible in culture, they unlike their parental hESC can undergo only a finite number of cell divisions before their growth is arrested and they senesce. Therefore there will be a need to constantly derive new batches of MSCs from hESCs to replenish the cell source of exosomes with each derivation necessitating recurring cost of derivation, testing and validation. To circumvent this need for re-derivation and ensure an infinite supply of identical MSCs for commercially sustainable production of exosomes as therapeutic agents or delivery vehicle, we explore the use of oncogenic transformation to bypass senescence. Oncogenic transformation could potentially alter the cell biology and affect the production or the properties of the exosomes. It was previously reported that transfection of *v-MYC *gene into fetal MSCs immortalized the cells but did not alter the fundament characteristics of these MSCs [30]. Here we transfected the MSCs with a lentiviral vector containing the *MYC *gene which encodes for the MYC protein into the previously described hESC derived MSCs (HuES9.E1 MSC) at passage 21 (p21) and passage 16 (p16) to generate a pooled cell line and three independently derived clonal cell lines respectively [26]. We examined the transformed cells according to the ISCT minimal defining criteria for MSCs namely plastic adherence, surface antigen profile of CD29^+^, CD44^+^, CD49a^+ ^CD49e^+^, CD90^+^, CD105^+^, CD166^+^, MHC I^+^, CD34^-^, CD45^- ^and HLA-DR^-^, and potential to differentiate into adipocytes, chondrocytes and osteocytes [31]. The secretion of these cells was evaluated for the presence of exosomes and the therapeutic efficacy of these exosomes were tested in a mouse model of MI/R as previously described [27].

## Methods

### Oncogenic transformation of HuES9.E1 MSC

The previously described human ESC-derived HuES9.E1 MSCs was infected at p21 or p16 with lentivirus carrying either a *MYC *gene or a *GFP *gene to generate two types of transfected cells, *MYC*-MSC and GFP-MSC, respectively. The *MYC *cDNA was amplified from pMXs-hc-MYC using primers PTDMYC (5' GAA TTC GAA TGC CCC TCA ACG TTA GC 3') and PTDMYCa (5' CTC GAG CGC ACA AGA GTT CCG TAG C 3') and cloned into pLVX-puro vector (Clontech, http://www.clontech.com) [32]. Lentiviral particles were produced using Lenti-X HT Packaging System and viral titer was determined by using a Lenti-X™ qRT-PCR titration kit (Clontech, http://www.clontech.com). The HuES9.E1 MSCs that were infected at p21 were plated at 10^6 ^cells per 10 cm dish and infected with viruses at a MOI = 5 in the presence of 4 μg/ml polybrene for overnight [26]. Cells were selected under 2 μg/ml puromycin for three days and expanded as per human ESC-derived HuES9.E1 MSCs and these cells were pooled to generate the E1-*MYC *21.1 line. For the HuES9.E1 MSCs that was infected at p16, three independently clonal lines (E1-*MYC *16.1, E1-*MYC *16.2 and E1-*MYC *16.3) were derived by limiting dilution [26]. When the cloned cells were expanded to 10^7 ^cells (or a confluent 15 cm culture dish), the passage number was designated as passage 1.

The cells were analyzed for *MYC *transgene integration, transcript and protein levels, surface markers, rate of cell cycling, telomerase activity, karyotype, genome-wide gene expression and differentiation potential (see additional file 1).

### HPLC purification of exosomes

The instrument setup consisted of a liquid chromatography system with a binary pump, an auto injector, a thermostated column oven and a UV-visible detector operated by the Class VP software from Shimadzu Corporation (Kyoto, Japan). The Chromatography columns used were TSK Guard column SWXL, 6 × 40 mm and TSK gel G4000 SWXL, 7.8 × 300 mm from Tosoh Corporation (Tokyo, Japan). The following detectors, Dawn 8 (light scattering), Optilab (refractive index) and QELS (dynamic light scattering) were connected in series following the UV-visible detector. The last three detectors were from Wyatt Technology Corporation (California, USA) and were operated by the ASTRA software. The components of the sample were separated by size exclusion i.e. the larger molecules will elute before the smaller molecules. The eluent buffer used was 20 mM phosphate buffer with 150 mM of NaCl at pH 7.2. This buffer was filtered through a pore size of 0.1 μm and degassed for 15 minutes before use. The chromatography system was equilibrated at a flow rate of 0.5 ml/min until the signal in Dawn 8 stabilized at around 0.3 detector voltage units. The UV-visible detector was set at 220 ηm and the column was oven equilibrated to 25°C. The elution mode was isocratic and the run time was 40 minutes. The volume of sample injected ranged from 50 to 100 μl. The hydrodynamic radius, Rh was computed by the QELS and Dawn 8 detectors. The highest count rate (Hz) at the peak apex was taken as the Rh. Peaks of the separated components visualized at 220 ηm were collected as fractions for further characterization studies.

### Testing secretion for cardioprotection

The conditioned medium was prepared by growing the transformed MSCs in a chemically defined serum free culture medium for three days as previously described [33]. The concentrated conditioned medium was processed by HPLC fractionation to obtain the exosomes as mentioned above. The exosomes were tested in a mouse model of MI/R injury. Myocardial ischemia was induced by 30 minutes left coronary artery (LCA) occlusion and subsequent reperfusion. Five minutes before reperfusion, mice were intravenously infused with 200 μl saline solution of 0.3 μg exosome protein purified from culture medium conditioned by *MYC*-MSCs. Control animals were infused with 200 μl saline. After 24 hours reperfusion, infarct size (IS) as a percentage of the area at risk (AAR) was assessed using Evans' blue dye injection and TTC staining as described previously [27]. All animal experiments were performed in accordance with the national guidelines on animal care and with prior approval by the Animal Experimentation Committee of Utrecht University.

### Statistical analysis

Two-way ANOVA with post-hoc Dunnett was used to test the difference in infarct size between groups. Correlation coefficient of each pairs of array was assessed using Pearson correlation test.

## Results

### Transforming HuES9.E1 MSC cultures

**Figure 1:**
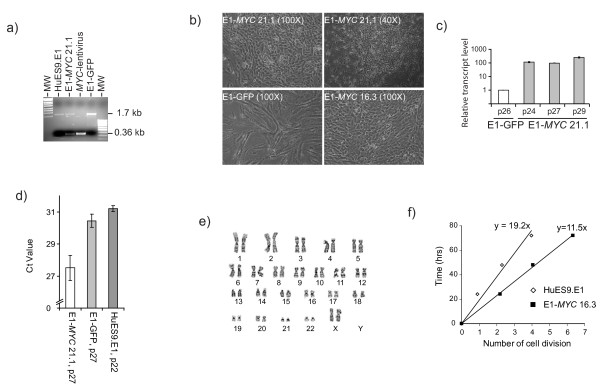
**Transformation of hESC-MSC**. (a) PCR analysis of cellular DNA from *MYC*-transfected HuES9.E1 MSCs (E1-*MYC *21.1), *GFP *transfected HuES9.E1 MSCs (E1-*GFP*) and the parental MSCs, HuES9.E1 (E1). DNA was amplified using primers specific for *MYC *exon 2 and exon 3, respectively. The expected PCR fragment size for the endogenous *MYC *gene was1.7 kb and for the transfected *MYC *cDNA was 0.36 kb as represented by the amplified fragment from the *MYC*-lentivirus. (b) Cell Morphology of transfected MSCs as observed under light microscopy. (c) Quantitative RT-PCR was performed on RNA from different passages of E1-*MYC *21.1 and GFP-MSCs for the level of *MYC *and *ACTIN *mRNA. The relative *MYC*-transcript level was normalized to that in GFP-MSCs. (d) Relative telomerase activity. 1 μg of cell lysate protein was first used to extend a TS primer by telomerase activity and the telomerase product was then quantitated by real time PCR. The Ct value represented the amount of telomerase product and was therefore indirectly proportional to telomerase activity in the lysate. (e) Karyotpye analysis of E1-*MYC *16.3 by G-banding. (f) Rate of cell cycling. Cells were labelled with CFDA and their fluorescence was monitored over time by flow cytometry. The loss of cellular fluorescence at each time point was used to calculate the number of cell division that the cells have undergone as described in Materials and Methods (Additional files 1).

HuES9.E1 MSCs at p 21 were infected with either *GFP*- or *MYC*-containing lentivirus. The infected cultures were placed under the puromycin selection for three days. Surviving cells were pooled. PCR amplification of genomic DNA demonstrated that the *MYC *transgene was successfully integrated in the genome (Figure 1a). Unlike the *MYC*-transfected cells which was pooled to form the E1-*MYC *21.1 line, the GFP-transfected cells progressed into senescence with decreasing rate of proliferation and acquiring a much flattened, spreading morphology (Figure 1b) and could not be propagated more than five passages post-transfection. The *MYC*-transformed cells expressed a 100 fold increase in *MYC *transcript level relative to the GFP-transfected cells (GFP-MSCs) (Figure 1c) and higher telomerase activity (Figure 1d). To generate independently cloned lines, three HuES9.E1 MSC cultures at p16 were independently transfected and placed under puromycin drug selection. The surviving cell cultures were cloned by limiting dilution to generate three lines, E1-*MYC *16.1, E1-*MYC *16.2 and E1-*MYC *16.3 lines, respectively. The lines were karyotyped by G-banding. The cell morphology of all three cell lines was similar to that of E1-*MYC *21.1 line. Only E1-*MYC *16.3 line had the parental karyotype of 46 XX with a pericentric inversion of chromosomal 9 between p11 and q13 in 20/20 metaphases, and was therefore used in all the subsequent experiments (Figure 1e) [26]. In contrast to their parental cells, the *MYC*-transformed cells proliferated faster with a population doubling time of 13 hours versus a population doubling time of 19 hours in untransformed MSCs. The average cell cycle time as measured using CFDA cell labelling as previously described was decreased from 19 hours to 11 hours (Figure 1f) [34]. The transformed cells effectively bypassed senescence and continued to maintain their proliferative rates for at least another 20 passages. The transformed cells were smaller and rounder in shape with prominent nuclei. At high cell density, these cells lose contact inhibition resulting in the formation of cell clusters (Figure 1b). Consistent with increased proliferation, the cells had higher levels of telomerase activity than *GFP*-transfected or non transfected cells (Figure 1d).

### Assessment of *MYC*-MSCs

**Figure 2:**
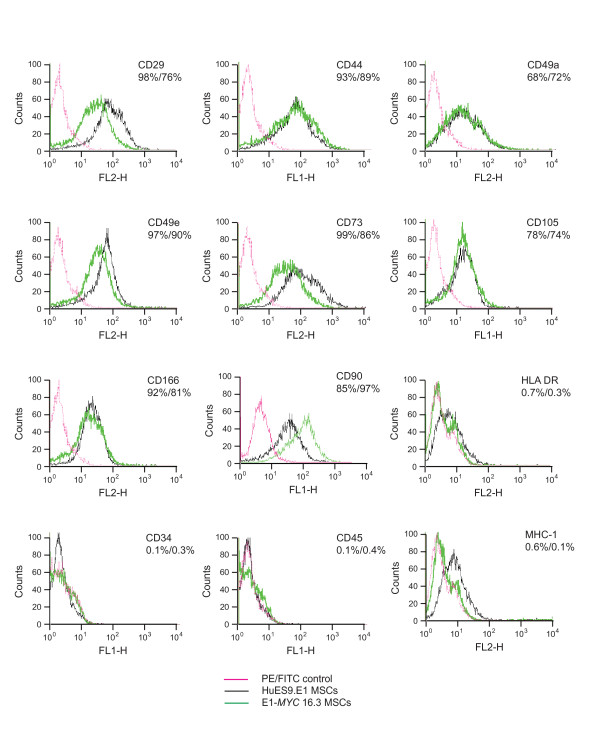
**Surface antigen profiling**. HuES9.E1 and E1-*MYC *16.3 MSCs were stained with an appropriate antibody conjugated to a fluorescent dye and analyzed by FACS. The fluorescence of HuES9.E1 or E1-*MYC *16.3 was the average cellular fluorescence of cells at p16 or p6. Nonspecific fluorescence was assessed by incubating the cells with isotype-matched mouse monoclonal antibodies.

**Figure 3:**
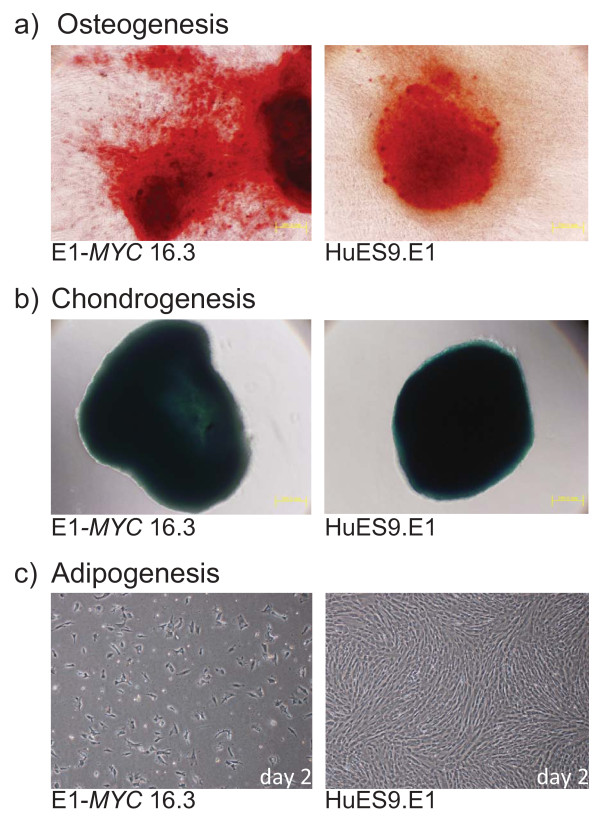
**Differentiation of HuES9E1 and E1-*MYC *16.3 MSCs**. MSCs were induced to undergo a) osteogenesis and then stained with von Kossa stain; b) chondrogenesis and then stained with Alcian blue; c) adipogenesis where E1-*MYC *16.3 and HuES9E1 MSCs were exposed to adipogenesis induction medium for two days. The cells were viewed at 100 × magnification.

The *MYC*-MSC culture were assessed according to the ISCT minimal criteria for the definition of human MSCs [31]. As observed earlier (Figure 1b), the culture did not adhere to plastic culture dishes as well as their untransformed MSCs especially at confluency when the cells started to form clusters instead of adhering to the plastic dish as a monolayer. The surface antigen profile of the *MYC*-transformed cells was quite similar to that of their parental cells except in their negative expression of MHC I. The cells were CD29^+^, CD44^+^, CD49a^+ ^CD49e^+^, CD73^+^, CD90^+^, CD105^+^, CD166^+^, MHC I^-^, HLA-DR^-^, CD34^- ^and CD45^- ^(Figure 2). The *in vitro *differentiation potential of both polyclonal E1-*MYC *21.1 and monoclonal E1-*MYC *16.3 cell lines was next examined (Figure 3). Both cell lines differentiated readily into chondrocytes and osteocytes (Figure 3a, b) but not adipocytes. The induction of adipogenesis in MSCs required 4 cycles of a 6-day treatment protocol consisting of 3 days' exposure to induction medium and 3 days' exposure to maintenance medium. We observed that exposure to the induction medium induced death in the *MYC*-transformed cells but not the untransformed parental cells (Figure 3c). These observations suggested that *MYC*-transformed cells cannot undergo adipogenic differentiation. Together, these observations demonstrated that unlike a previous report where *MYC *transformation was observed not to alter the fundamental characteristics of MSCs, we observed here that *MYC *transformation affected a defining property of MSCs i.e. the potential to undergo adipogenesis [30].

### Gene expression profile

**Figure 4:**
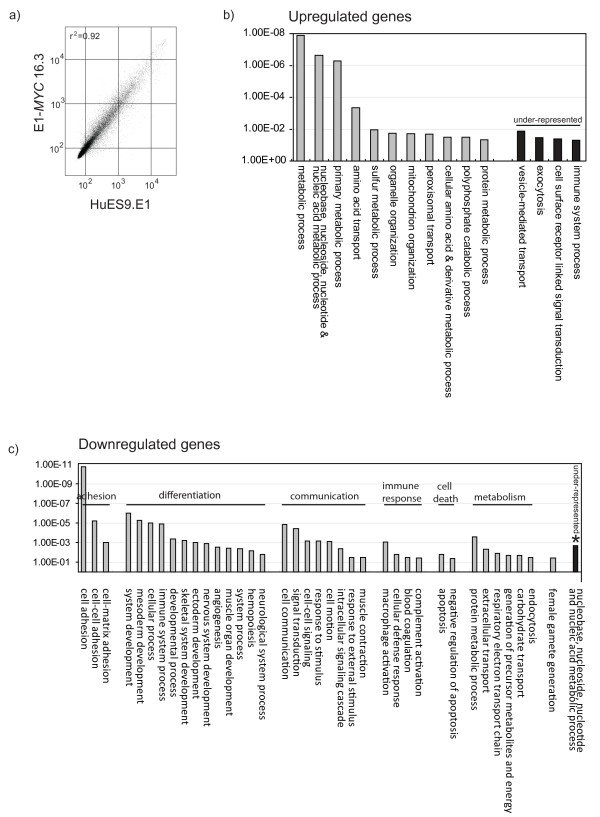
**Gene expression analysis**. RNA from HuES9E1 and E1-*MYC *16.3 MSCs were hybridized to Sentrix HumanRef-8 Expression BeadChip Version 3 and analyzed by Beadstudio and Genespring GX 10. a) Pairwise comparison of gene expression between HuES9.E1 and E1-*MYC *16.3 MSCs using Beadstudio analysis. b,c) PANTHER analysis. 161 genes that were over-expressed by > 2-fold and 226 under-expressed genes that were under-expressed by > 2-fold in E1-*MYC *16.3 MSCs were analyzed using PANTHER algorithm. The observed frequency of genes for each biological process in each gene set was compared with the reference frequency which, in this case is the frequency of genes for that biological process in the NCBI database. Those biological processes whose observed frequency exceeds the reference frequency with a p < 0.05 are considered significant.

Genome-wide gene expression profiling of *MYC*-transformed MSCs and their parental MSCs by microarray hybridisation was performed to assess the relatedness between the cell types. Microarray hybridization was performed in duplicate on Sentrix Human Ref-8 Expression BeadChip using RNA from E1-*MYC *16.3 MSCs at p4, p7, and p8, and from the parental HuES9-E1 MSCs at p15 and p16. The gene expression profile (Accession number: GSE25296) among different passages of E1-*MYC *16.3 MSCs or among different passages of the parental HuES9-E1 MSCs was highly similar with a correlation coefficient, r^2 ^being greater than 0.98. The correlation coefficient, r^2 ^between E1-*MYC *16.1 MSCs and parental HuES9-E1 MSCs, was also relatively high at 0.92 (Figure 4a). A total of 161 genes was upregulated and 226 genes downregulated at least 2 fold in E1-*MYC *16.1 MSCs suggesting that there were changes in gene expression after *MYC *transformation. These differentially expressed genes were functionally clustered by PANTHER (Protein ANalysis THrough Evolutionary Relationships) in which the observed frequency of genes for each biological process in each gene set was compared with the reference frequency which, in this case is the frequency of genes for that biological process in the NCBI database [35,36]. There were 11 over-represented biological processes for the 161 upregulated genes namely, metabolic process, nucleobase, nucleoside, nucleotide and nucleic acid metabolic process, primary metabolic process, amino acid transport, sulfur metabolic process, organelle organization, mitochondrion organization, peroxisomal transport, cellular amino acid and derivative metabolic process, polyphosphate catabolic process and protein metabolic process. There were 4 under-represented processes: vesicle-mediated transport, exocytosis, cell surface receptor linked signal transduction, and immune system process (Figure 4b). In the 226 downregulated genes, there were 37 over- and 1 under-represented biological processes (Figure 4c).

For the up-regulated genes, many of the associated over-represented processes were generally important for increasing cell mass or anabolic activity for cell division and were consistent with the observed increased cell proliferation activity. The under-represented processes, namely vesicle-mediated transport, exocytosis suggested that exosome production might not be affected. For the down-regulated genes, the 37 over-represented processes could be broadly classified into processes that are associated with adhesion, differentiation, communication, immune response, cell death and metabolism. These processes were also consistent with some of our observations of the *MYC*-transformed MSCs, namely reduced adherence to plastic, loss of adipogenic differentiation potential and loss of MHC I expression.

### Cardioprotective activity of secretion

**Figure 5:**
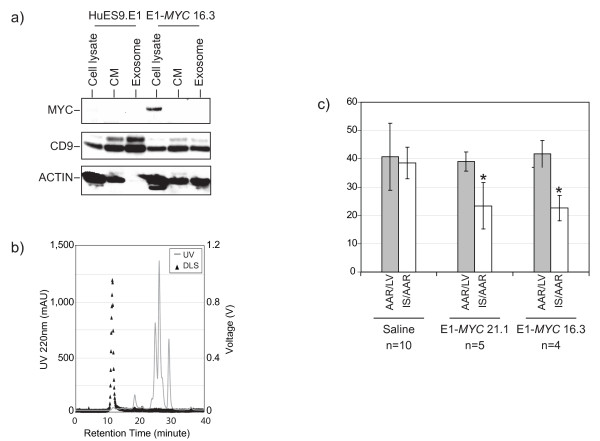
**Analysis of secretion**. (a) Western blot analysis. Proteins from cell lysate, conditioned medium (CM), and HPLC purified exosomes of E1MSCs or E1-*MYC*-MSCs were separated on SDS-PAGE and probed with different antibodies to detect MYC (64 kDa), ACTIN (42 kDa), and CD9 (24 kDa). (b) HPLC fractionation and dynamic light scattering of CM from E1-*MYC*-MSC. CM was fractionated on a HPLC using BioSep S4000, 7.8 mm × 30 cm column. The components in CM were eluted with 20 mM phosphate buffer with 150 mM of NaCl at pH 7.2. The elution mode was isocratic and the run time was 40 minutes. The eluent was monitored for UV absorbance at 220 ηm. Each eluting peak was then analyzed by light scattering. The fastest eluting peak (arrow) was collected for testing in a mouse model of myocardial ischemia/reperfusion injury. (c) 0.3 μg HPLC-purified exosomes was administered intravenously to a mouse model of acute myocardial/ischemia reperfusion injury five minutes before reperfusion. Infarct size (IS) as a percentage of the area at risk (AAR) upon treatment with saline (n = 10), exosomes from E1-*MYC *21.1 (n = 5) and exosomes from E1-*MYC *16.3 (n = 4) were measured. The relative infarct size (IS/AAR) in mice treated with E1-*MYC *21.1 exosome or E1-*MYC *16.3 exosome was 23.4 ± 8.2%, and 22.6 ± 4.5%, respectively and their relative infarct sizes were significantly lower than the relative infarct size of 38.5 ± 5.6% in saline-treated mice (p < 0.001 and p < 0.002, respectively).

The loss of adipogenic potential in *MYC*-transformed MSC suggested that other aspects of the characteristics of ESC-derived MSCs such as the production of therapeutic exosomes might also be compromised by the transformation. We had previously demonstrated that exosomes secreted by ESC-derived MSCs was protective in a mouse model of MI/R injury [27]. To test if this aspect was compromised, the transformed cells were grown in a chemically defined medium, the conditioned culture medium harvested and exosomes were purified as previously described [24,33]. Despite increased *MYC *transcript and protein levels in the transformed cells, MYC protein was not detectable in the conditioned medium and purified exosomes (Figure 5a). The HPLC protein profile of the conditioned medium was similar to that of conditioned medium from untransformed MSCs (Figure 5b) with the fastest eluting fraction having a retention time of about 12 minutes [24]. Dynamic light scattering analysis of this peak revealed the presence of particles that were within a hydrodynamic radius range of 50-65 ηm. In a typical run, we routinely purified about 1.5 mg of exosomes per liter of conditioned medium. HPLC-purified exosomes from either E1-*MYC *21.1 or E1-*MYC *16.3 was administered to the mouse model of MI/R injury at a dosage of 0.3 or 0.4 μg per mouse respectively (Figure 5c). The area at risk (AAR) as a percentage of left ventricular (LV) area in E1-*MYC *21.1 exosome, E1-*MYC *16.3 exosome, or the saline-treated control group was similar at 39.1 ± 3.4% (n = 5), 41.7 ± 4.7% (n = 4) and 40.8 ± 11.8% (n = 10), respectively. The relative infarct size (IS/AAR) in mice treated with E1-*MYC *21.1 exosome or E1-*MYC *16.3 exosome was 23.4 ± 8.2%, and 22.6 ± 4.5%, respectively and their relative infarct sizes were significantly lower than the relative infarct size of 38.5 ± 5.6% in saline-treated mice (p < 0.001 and p < 0.002, respectively).

## Discussion

This report describes the transformation of human ESC-derived MSCs by over-expression of *MYC *gene. This transformation enabled the cells to bypass senescence, increase telomerase activity and enhance proliferation. Generally, genome-wide gene expression between the transformed cells versus their parental cells was conserved with a correlation coefficient of 0.92. The transformed cells also have the characteristic surface antigen profile: CD29^+^, CD44^+^, CD49a^+^, CD49e^+^, CD90^+^, CD105^+^, CD166^+^, MHC I^-^, HLA-DR^-^, CD34^- ^and CD45^-^. Although the transformed cells fulfilled most of fundamental requisites in ISCT minimal criteria for the definition of human MSCs, they nevertheless have an altered MSC phenotype [31]. They exhibited reduced adherence to plastic and failed to undergo adipogenesis which ironically was reported to be most robust among the three fundamental MSC differentiation potentials in the human ESC-derived MSCs [26]. Therefore, in contrast to a previous report that observed no fundamental changes in MSC properties after *MYC *transformation, we observed some fundamental changes in *MYC*-transformed cells such that the cells no longer fulfilled the ISCT minimal criteria for the definition of human MSCs and are technically not MSCs [30,31]. Despite the loss of a defining MSC property, the *MYC*-transformed cells continued to secrete exosomes that could reduce infarct size in a mouse model of MI/R injury. The relative infarct size was 23.4 ± 8.2% and 22.6 ± 4.5% in mice treated with exosomes from the polyclonal and monoclonal lines, respectively. The relative infarct size in saline treated mice was 38.5 ± 5.6%. These relative infarct sizes were comparable to those observed in mice treated with exosomes from the untransformed parental MSCs or fetal MSCs [24,25]. The relative infarct sizes in mice treated with these exosomes were 17.0 ± 3.6% and 18.1 ± 2.0%, respectively against a 34.5 ± 3.3% in saline treated mice. Therefore, both independently transformed polyclonal and monoclonal lines also produced exosomes with similar therapeutic efficacy as those produced by untransformed MSCs indicating that exosome production was independent of the transformation and was consistent and reproducible. The significant reduction of infarct size by exosome treatment and the well established correlation between infarct size and subsequent adverse remodeling suggests that exosome treatment would enhance the prognostic outcome of reperfusion therapy [37]. We noted that MYC protein was present in the transformed cells but was not detectable in either the conditioned medium or exosome. As onco-protein unlike oncogene cannot be replicated or amplified, the risk of tumorigenesis by exosomes from *MYC*-transformed cells is further mitigated. The use of lentiviral vectors for the transformation of the cells poses another potential safety risk. Since the secreted exosome and not the transformed cells will be used as therapeutic agents, the risk from the integration of lentivirus is mitigated. Also the use of newer generation of lentiviral vector which in our case is a third generation lentiviral vector further reduces the risk of producing infectious recombinant viral particles. For the actual manufacture of therapeutic exosomes, we propose transforming the cells using some of the lentiviral vectors that are currently being tested in clinical trials [38]. This will further reduce the risks associated with the use of lentiviral vectors for transformation.

## Conclusion

In summary, *MYC *transformation represents a practical strategy in ensuring an infinite supply of cells for the production of exosomes in the milligram range as either therapeutic agents or delivery vehicles. In addition, the increased proliferative rate reduces the time for cell production and thereby reduces production costs. In conclusion, this work despite the lack of exciting novel scientific insights into biological processes provides a critical enabling technology for the development of a cost effective production process for consistent supplies of HPLC-purified therapeutic human exosomes.

## List of abbreviations

MSC: Mesenchymal Stem cells; ESC: Embryonic stem cells; MI/R: myocardial ischemia/reperfusion

## Competing interests

The authors declare that they have no competing interests.

## Authors' contributions

SKL conceived the idea. TSC and SKL wrote the paper, designed the experiments, interpreted the data; TSC, FA., YY, SST, RCL. and JP performed the experiments; DdK, AC, and CNL contributed to the discussion of the experimental design and interpretation of data. All authors have read and approved the final manuscript.
